# Impact of Cognitive Profile on Impulse Control Disorders Presence and Severity in Parkinson's Disease

**DOI:** 10.3389/fneur.2019.00266

**Published:** 2019-03-22

**Authors:** Alice Martini, Luca Weis, Eleonora Fiorenzato, Roberta Schifano, Valeria Cianci, Angelo Antonini, Roberta Biundo

**Affiliations:** ^1^School of Psychology, Keele University, Newcastle-under-Lyme, United Kingdom; ^2^IRCCS San Camillo Hospital, Venice, Italy; ^3^Department of Neuroscience (DNS), University of Padua, Padua, Italy

**Keywords:** Parkinson's disease, mild cognitive impairment, dementia, impulse control disorder, cognitive profile, cognition, cognitive states, cognitive phenotypes

## Abstract

**Background:** Impulse control disorders (ICDs) and related behaviors are frequent in Parkinson's disease (PD). Mild cognitive impairment (PD-MCI) and dementia (PDD), both characterized by heterogeneous cognitive phenotypes, are also commonly reported in PD. However, the frequency and severity of ICD within PD cognitive states is unknown.

**Methods:** Three hundred and twenty-six PD patients completed a comprehensive neuropsychological assessment and were classified as PD-MCI, PDD, or without cognitive alterations (PD-NC). The Minnesota impulsive disorders interview was used to ascertain the presence (ICD+) or absence (ICD–) of ICD. The Questionnaire for Impulsive-Compulsive Disorders in Parkinson's Disease-Rating Scale was used to assess ICD severity. A subsample of 286 patients evaluated with the same cognitive tasks was selected in order to investigate the characteristics of ICD in PD cognitive phenotypes.

**Results:** ICDs were present in 55% of PD-NC, in 50% of PD-MCI, and in 42% of PDD patients. Frequencies of ICD+ with attentive (ICD+: 20% vs. ICD–: 4%; *p* = 0.031) and executive impairments (ICD+: 44% vs. ICD–: 30%; *p* = 0.027) were higher in the PD-MCI and PDD subgroups, respectively. As expected, no differences were observed in the PD-NC. PD-MCI with attentive impairments presented higher percentage of ICD+ with deficits in the Trail Making Test B-A but not in the Digit Span Sequencing task. In PDD, executive failures concerned Similarities task (ICD+: 67%; ICD–: 29%; *p* = 0.035), with no differences between ICD+ and ICD– in the Stroop task.

**Conclusions:** Prevalence and severity of ICDs and related behaviors do not differ in PD with different cognitive states. However, ICD+ are more likely to show deficits, respectively in attentive and in executive domains, specifically in the Trail Making Test B-A task for the attention and working memory domain in PD-MCI and in the Similarities task for the executive function domain in PDD. Prospective studies should evaluate if these tests can be used as screening tool for ICDs in PD.

## Introduction

In Parkinson's disease (PD), impulse control disorders (ICDs) are reported in around 30% of medicated patients (1, 2). They include pathological gambling (PG), hypersexuality (HS), compulsive shopping (CS), and binge-eating (BE). Either alone or in co-occurrence with the major ICDs, other repetitive and compulsive behaviors have been observed (3, 4). These are referred as impulsive-compulsive behaviors (ICBs) and include punding (repetitive simple non goal-oriented behaviors), hobbyism (repetitive complex behavior), and dopamine dysregulation syndrome (DDS), which is a pattern of compulsive dopaminergic medication use.

Prevalence rates of ICDs are similar in drug naïve PD patients and in the general population (5, 6), but higher in medicated PD patients (1, 2). The association between dopaminergic medications and ICDs is now well-recognized (7), with an increased risk for PD patients taking dopamine agonists alone or together with levodopa (1, 8).

In addition to dopaminergic therapy, other demographic and clinical variables may interact with exogenous and endogenous dopaminergic levels, therefore increasing the susceptibility to ICDs (8–10). Moreover, patients with ICDs report higher rates of anhedonia (11), depression and anxiety (2, 12, 13), and cognitive impairments (14, 15).

Cognitive deficits are common in PD and a significant proportion is at risk to develop dementia (PDD) (16). Evidence suggests that mild cognitive impairment in PD (PD-MCI) is a frequent condition (17) and refers to a state of cognitive alterations but preserved daily living autonomy, therefore representing an intermediate stage between normal cognition and dementia (18, 19). PD-MCI is characterized by heterogeneous cognitive profile (20, 21) and cognitive phenotypes may be differently associated with the presence and severity of specific non-motor symptoms, possibly underlying pathophysiological variability (22).

Both PD-MCI and PDD as well as ICD are well-recognized cognitive and behavior conditions in PD. Since patients with PD normal cognition (PD-NC), PD-MCI, and PDD differ for demographic and clinical features, we might expect ICD prevalence and characteristics to differ between these cognitive categories. For example, in PDD the use of dopamine agonists is discouraged due to the likelihood to develop psychosis (23, 24) which in turn might result in reduced risk of ICD. Younger age is one of the risk factors for ICD in PD (8), possibly related to preserved ventral striatal responsiveness and dopaminergic overstimulation (25). By contrast, PDD, who are older than PD-NC and PD-MCI, might be less susceptible to ICD. This concept would be also supported by a previous study showing lower prevalence rates of dementia in patients with vs. without ICD (26).

A recent meta-analysis showed worse performance of PD patients with ICD in set-shifting and reward-related decision-making tasks (15). To our knowledge, there are no studies on ICDs prevalence across cognitive states and specific domains. This is an important issue as recognizing factors associated with ICD in PD across cognitive states and domains may improve clinical diagnosis and pave the way for future studies on therapeutic management. Considering the heterogeneous cognitive profile disclosed by PD patients, we might expect that ICD rates would change according to the cognitive domains affected.

Here, for the first time, ICDs and related behaviors will be described across PD patients with normal cognition (PD-NC), PD-MCI, and PPD, and within specific cognitive phenotypes. The study aims to investigate whether PD cognitive states and phenotypes are associated with changes in prevalence and severity of ICDs.

## Materials and Methods

### Patients and Clinical Assessment

**Figure 1:**
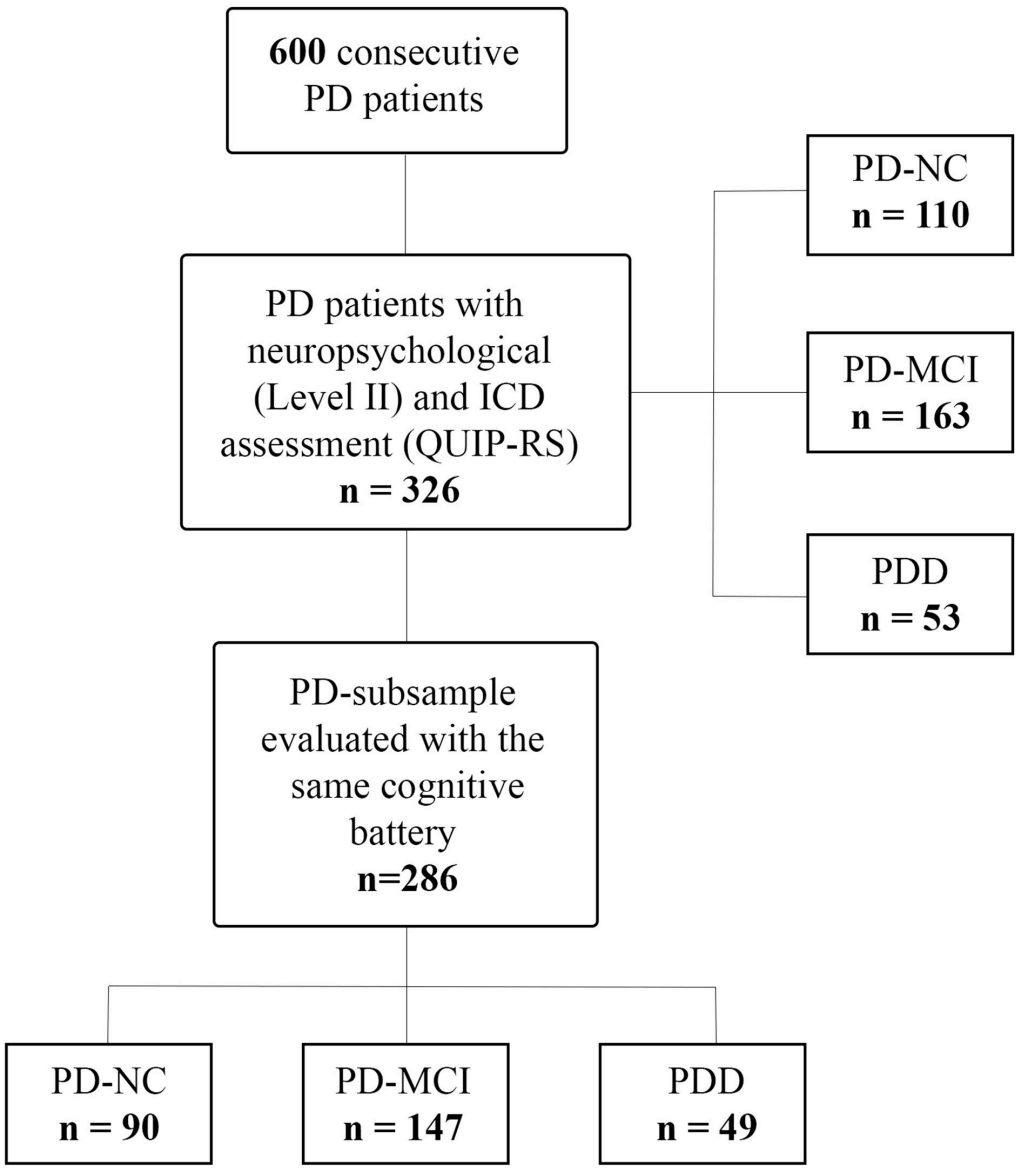
Study flowchart. PD, Parkinson's disease; PD-NC, PD with normal cognition; PD-MCI, PD with mild cognitive impairment; PDD, PD with dementia; ICD, impulse control disorder; QUIP-RS, Questionnaire for Impulsive-Compulsive Disorders in Parkinson's Disease-Rating Scale.

We recruited 600 consecutive patients with PD at the Parkinson's disease and Movement Disorders Unit, Neurology Clinic in Padua, Italy, and IRCCS San Camillo Hospital in Venice, between May 2010 and August 2018. All patients met the clinical diagnostic criteria of the UK Parkinson's Disease Society Brain Bank (27). Exclusion criteria were diagnosis of atypical Parkinsonism as well as clinically significant or unstable medical conditions including cardiovascular, metabolic, psychiatric diseases and neurosurgical procedures (including deep brain stimulation). Among this large cohort, we included only PD patients who underwent a comprehensive neuropsychological evaluation according to Level II criteria (28, 29), and ICD assessment with Minnesota Impulsive Disorder Interview (MIDI) and the Questionnaire for Impulsive-Compulsive Disorders in Parkinson's Disease-Rating Scale (QUIP-RS) (30), resulting in a sample of 326 PD patients (see Figure 1). Of note, diagnosis of ICDs and ICBs were based on the MIDI, which was administered by an experienced neuropsychologist. ICDs and ICBs that were not included in the MIDI but were already well-known to occur in the PD population were also investigated, namely BE, punding, and DDS. All patients diagnosed with ICDs answered affirmatively one gateway question plus an affirmative answer to one or more of the remaining questions. In order to evaluate ICDs severity, the QUIP-RS was also administered. Finally, single and multiple ICDs and ICBs prevalence rates were also investigated using published QUIP-RS cutoffs (30), following a previous study of PD patients with ICDs in Italian cohorts (31).

Demographic information including sex, age, education, age at symptoms onset, disease duration, and dopaminergic medication were also collected. We calculated dopamine agonist equivalent daily dose (DAED) and total L-dopa-equivalent daily dose (LEDD) for each patient according to Tomlinson et al. (32); further, DAED and LEDD were adjusted by body weight (DAED/kg and LEDD/kg). Disease severity was assessed with the motor part of the Movement Disorder Society Unified Parkinson's Disease Rating Scale (MDS-UPDRS-III) (33).

All subjects underwent a comprehensive assessment including functional autonomy (by instrumental- and activity of daily living, ADL/IADL) (34), subjective cognitive complaints and their impact on daily functioning (by the Parkinson's Disease—Cognitive Functional Rating Scale, PD-CFRS) (35) and presence of depression, anxiety and the quality of life using the Beck Depression Inventory (BDI-*II*), State-Trait Anxiety Inventory forms (STAI-Y1 and Y2), and an 8-item version of the Parkinson's Disease Questionnaire for quality of life (PDQ-8), respectively (36).

Patients underwent a comprehensive neuropsychological battery as previously described (17), specifically designed to target cognitive deficits in Parkinson's disease with at least two tests for each cognitive domain (e.g., attention and working memory, executive, memory, language, and visuospatial abilities) (28, 29).

We calculated z-scores for each test and participant, based on standardized published Italian norms that are adjusted for age and education, then PD patients were classified as PD-MCI if z-score was at least 1.5 SD below appropriate norms on at least two tests (i.e., within a single cognitive domain or at least one test in two or more cognitive domains) (28). Presence of PDD was assessed based on the Movement Disorders Society Task force recommendations (29), which included cognitive, daily functioning, and behavioral assessment. Patients without cognitive alterations were defined as PD-NC. Neuropsychological tests were performed on two separate occasions within 5–7 days and administered in the morning ON medication.

Finally, to investigate the association between presence of ICDs and cognitive phenotypes, we selected a PD-subsample, which was evaluated with the same cognitive battery, leaving a final sample of 286 PD (see Figure 1).

Specifically, attention and working memory domain was tested with the Trail Making Test part B-A (TMT B-A) (37) and Digit Span Sequencing (DSS) of Wechsler Adult Intelligence Scale–Fourth Edition (WAIS–IV) (38). Executive functions were evaluated with the Stroop Color and Word test (39), and the WAIS-IV similarities (38). Memory was assessed with the delayed recall of Rey-Osterrieth complex figure test (ROCF) (40), and prose memory tests (41). Language was tested with the semantic fluency task, and Novelli's naming test (42). Visuospatial and visuoperceptive functions were assessed by Benton's Judgment of Line Orientation Test (43), and the Visual Object and Space Perception incomplete letters recognition subtask (44).

Patients gave written informed consent, according to the Declaration of Helsinki, before study enrolment, and ethical approval was obtained from the Venice Research Ethics Committee, Venice, Italy.

### Statistical Analysis

Statistical analyses were performed using SPSS version 22 (IBM SPSS, Chicago, IL) (45). Demographic and clinical continuous variables were analyzed using Kruskal-Wallis test, with Mann–Whitney-*U post-hoc* test (*p* < 0.05) for between-groups comparisons. Pearson's Chi-square test was applied to categorical variables. Frequencies of ICDs and related behaviors across cognitive states were investigated using Pearson's Chi-square test. Linear trend of increase/decrease in frequency by cognitive decline status was investigated using Chi-square test for trend. ICDs severity and related behaviors across cognitive states were compared between groups via ANCOVA model including the continuous QUIP-RS score as dependent variable and as covariate those demographic and clinical variables differing between cognitive states, which has a significant effect on the QUIP-RS continuous score in a multiple regression model. Distribution normality was checked with Kolmogorov–Smirnov tests and homogeneity of variance with Levene's test.

Within each cognitive state, the frequencies of PD failing two tests of the same cognitive domain were compared between patients with (ICD+) and without (ICD–) ICDs/ICBs, using Pearson's Chi-square test. For all analyses, the significance threshold was set at *p* < 0.05.

## Results

### PD Demographic and Clinical Characteristics Among Cognitive States

Out of 326 PD patients, 110 were cognitive normal (PD-NC), 163 had MCI (PD-MCI) and 53 dementia (PDD).

Mean age was different across subgroups (PDD>PD-MCI>PD-NC, *p* < 0.0001) while gender distribution was similar. PD-NC had lower age at symptoms onset, and higher years of education than both PD-MCI and PDD groups (*p* < 0.0001 for both variables). PDD had longer disease duration compared to PD-NC and PD-MCI groups (*p* = 0.006).

The three PD cognitive subgroups did not differ for LEDD and LEDD/kg. However, the DAED, the DAED/kg, and the percentage of patients under DA were lower in the PDD group compared to PD-NC and PD-MCI groups (*p* = 0.0002, *p* = 0.0001, *p* = 0.0005, respectively).

UPDRS-I and UPDRS-II scores were higher in the PDD, but comparable in the PD-NC and PD-MCI (*p* = 0.0002 and *p* < 0.0001, respectively). The UPDRS-III scores were different across the three subgroups, with the lowest scores in PD-NC, and the highest in PDD (*p* < 0.0001). Global cognitive status (measured by mean of MMSE and MoCA scales) was different across the three subgroups, with best cognitive performances observed in PD-NC and worst in PDD (*p* < 0.0001 for both variables). BDI-*II* scores differed across the three subgroups, with the lowest value in PD-NC and the highest in the PPD (*p* < 0.0001). However, the percentage of patients with BDI-*II* score above the cutoff (>14) was higher in PDD (*p* = 0.0027), but comparable in the PD-NC and PD-MCI. State (STAI-Y1), but not trait (STAI-Y2) anxiety score, was higher in PDD compared to PD-NC and PD-MCI (*p* = 0.0076). PDD had greater disability on ADL/IADL compared to PD-NC and PD-MCI groups (*p* < 0.0001 and *p* < 0.0002, respectively). Finally, functional disability due to mainly cognitive impairments (PD-CFRS) was significantly different across PD cognitive subgroups (PDD>PD-MCI>PD-NC) (*p* < 0.0001). Demographic and clinical data are reported in Supplementary Table 1.

### Demographic and Clinical Characteristics Associated With ICD Among Cognitive States

Out of 326 PD patients, 60 PD-NC patients, 81 PD-MCI patients, and 22 PDD patients were diagnosed with presence of at least one ICD or ICB.

In PD-NC, ICD+, and ICD– did not differ for mean age, gender distribution, education level, and age at symptoms onset, although ICD+ had longer disease duration (*p* = 0.0017). LEDD and LEDD/Kg were higher in the ICD+ (*p* = 0.0002 and *p* = 0.0001, respectively), but there were no differences in the DAED, DAED/Kg, and in the percentage of patients under DA.

In PD-MCI, ICD+ had lower mean age and age at symptoms onset, and longer disease duration than ICD– (*p* = 0.0142, *p* < 0.0001, and *p* = 0.0003, respectively). LEDD, LEDD/Kg, DAED, DAED/Kg, and the percentage of patients under DA were higher in the ICD+ compared to ICD– (*p* = 0.0028, *p* = 0.0156, *p* = 0.0305, *p* = 0.0469, and *p* = 0. 0013, respectively).

In both PD-NC and PD-MCI, the quality of life of ICD+ patients was worse (*p* = 0.0009 and *p* = 0.0052, respectively). Conversely, UPDRS-I, UPDRS-II, and UPDRS-III scores, global cognitive status (measured by MMSE and MoCA scales), BDI-*II* score and percentage of patients with BDI-*II* score above the cutoff, state and trait anxiety (STAI-Y1 and STAI-Y2 scores), disability on the ADL, IADL, and PD-CFRS scales did not differ between ICD+ and ICD–.

In the PDD, there were no difference between ICD+ and ICD– in any demographic and clinical characteristic investigated. Demographic and clinical data of ICD+ and ICD– among cognitive states are reported in Table 1.

**Table 1:** Demographic and clinical characteristics of ICD+ and ICD– across PD cognitive states.

	**PD-NC** ** (*n*** **=** **110)**		**PD-MCI** ** (*n*** **=** **163)**		**PDD** ** (*n*** **=** **53)**		**ICD+** **vs. ICD-**		
	**ICD+** ***n* = 60**	**ICD–** ***n* = 50**	**ICD+** ***n* = 81**	**ICD–** ***n* = 82**	**ICD+** ***n* = 22**	**ICD–** ***n* = 31**	**PD-NC**	**PD-MCI**	**PDD**
Age (yr)	60.58 (9.36)	61.48 (10.49)	67.24 (8.61)	70.27 (8.93)	71.50 (9.39)	74.10 (7.53)	0.5991	**0.0142**	0.3199
Sex (%, male)	49%	59%	68%	55%	67%	71%	0.4007	0.1356	0.9812
Education (yr)	12.80 (3.82)	12.70 (4.35)	9.45 (4.45)	9.85 (4.46)	9.91 (4.85)	8.55 (4.65)	0.8657	0.4991	0.1901
Age of onset symptoms (yr)	51.45 (10.36)	54.56 (10.32)	55.55 (10.20)	62.67 (10.48)	59.22 (10.40)	63.17 (9.67)	0.1604	** < 0.0001**	0.2032
Disease duration (yr)	9.12 (4.54)	6.08 (5.45)	10.85 (6.55)	7.10 (5.09)	11.50 (5.19)	11.00 (5.15)	**0.0017**	**0.0003**	0.8432
LEDD	963.06 (476)	589.40 (507.28)	973.90 (492.03)	750.54 (526.12)	814.06 (416.35)	655.86 (380.37)	**0.0002**	**0.0028**	0.1405
LEDD/kg	14.00 (7.91)	8.10 (6.75)	13.45 (7.04)	11.00 (8.19)	10.98 (5.78)	8.76 (4.73)	**0.0001**	**0.0156**	0.1711
DA (%)	80%	73%	90%	67%	57%	52%	0.5684	**0.0013**	0.9418
DAED	157.37 (110.42)	132.16 (117.06)	141.46 (95.05)	110.30 (113.62)	79.95 (96.07)	78.15 (87.52)	0.2786	**0.0305**	0.8610
DAED/kg	2.29 (1.77)	1.86 (1.65)	1.97 (1.41)	1.65 (1.85)	1.06 (1.24)	1.03 (1.16)	0.3143	**0.0469**	0.8015
MDS-UPDRS-I	10.57 (5.41)	9.24 (5.51)	11.19 (4.89)	9.29 (4.44)	13.88 (7.47)	16.73 (7.57)	0.3944	0.0748	0.5597
MDS-UPDRS-II	11.67 (6.43)	9.43 (6.47)	14.29 (6.94)	11.62 (6.37)	19.50 (4.31)	19.47 (8.93)	0.1137	0.0972	0.7465
MDS-UPDRS-III	20.75 (12.64)	18.00 (12.76)	28.52 (11.72)	24.67 (12.84)	37.46 (10.38)	33.69 (13.06)	0.2177	0.0894	0.3347
ADL	5.74 (0.60)	5.83 (0.81)	5.43 (1.01)	5.35 (0.96)	4.39 (1.33)	3.52 (1.91)	0.1045	0.4780	0.1441
IADL	5.96 (1.44)	5.95 (1.66)	5.45 (1.66)	5.64 (1.64)	3.39 (1.58)	2.85 (1.81)	0.8489	0.3608	0.3357
PD-CFRS	2.24 (2.23)	1.42 (1.75)	4.61 (4.16)	3.40 (3.35)	10.88 (5.28)	13.87 (6.97)	0.1025	0.1495	0.2153
PDQ-8	9.60 (5.29)	5.80 (4.23)	10.93 (5.30)	8.47 (5.33)	12.56 (6.44)	14.18 (5.53)	**0.0009**	**0.0052**	0.3974
STAI-Y1	37.82 (11.47)	37.97 (8.58)	38.86 (10.58)	39.62 (10.09)	42.25 (11.05)	44.05 (8.98)	0.4616	0.6162	0.4636
STAI-Y2	41.79 (10.80)	41.00 (9.49)	41.25 (10.40)	41.58 (10.63)	44.94 (11.80)	45.45 (10.54)	0.7461	0.9260	0.7499
BDI-*II*	9.10 (8.02)	8.35 (6.60)	10.62 (7.05)	10.77 (8.26)	12.80 (7.06)	15.54 (7.40)	0.9235	0.7234	0.2616
BDI-*II* (%, cutoff > 14)	18%	16%	28%	28%	40%	54%	0.9775	0.8829	0.5263
MoCA	27.52 (2.06)	27.55 (1.86)	25.98 (2.82)	25.62 (2.18)	21.37 (4.30)	21.10 (4.36)	0.7254	0.2793	0.6006
MMSE	25.89 (2.39)	25.04 (2.65)	22.30 (3.52)	22.00 (2.95)	15.68 (4.85)	17.17 (3.66)	0.1248	0.5933	0.3682

*Significant differences (p < 0.05) are reported in bold type. SD, standard deviation; PD, Parkinson's disease; PD-NC, PD with normal cognition; PD-MCI, PD with mild cognitive impairment; PDD, PD with dementia; ICD+, patients with impulse control disorders and related behaviors according to MIDI; ICD-, patients without impulse control disorders and related behaviors according to MIDI; MDS-UPDRS, Movement Disorder Society Unified Parkinson's Disease Rating Scale; LEDD, levodopa equivalent daily dose; DAED, dopamine agonist equivalent dose; LEDD/kg, LEDD adjusted by body weight; DAED/kg, DAED adjusted by body weight; ADL, Activity of daily living; IADL, Instrumental activities of daily living; PD-CFRS, Parkinson's Disease—Cognitive Functional Rating Scale; PDQ-8, Parkinson's Disease Questionnaire; STAI (Y-1, Y-2), State-Trait Anxiety Inventory; BDI-II, Beck Depression Inventory-II; MoCA, Montreal Cognitive Assessment; MMSE, Mini Mental State Examination*.

### ICDs Presence and Severity Across Cognitive States

**Figure 2:**
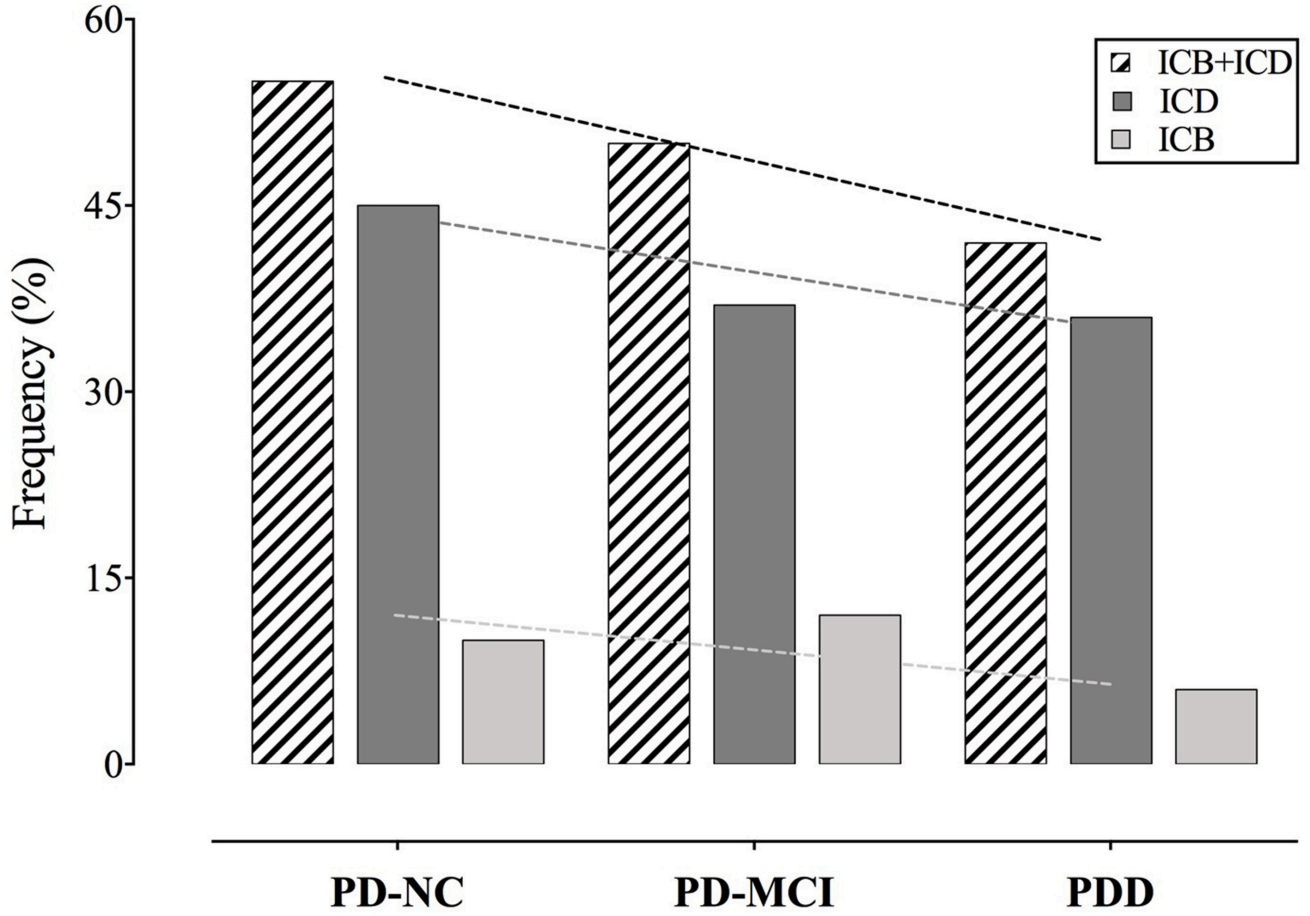
Frequency of ICDs and ICBs among cognitive states. Slopes of the trend lines are reported. PD, Parkinson's disease; PD-NC, PD with normal cognition; PD-MCI, PD with mild cognitive impairment; PDD, PD with dementia; ICD, impulse control disorder; ICB, impulsive compulsive behavior.

According to the MIDI, ICDs, and/or ICBs were present in 55% (60 patients) of PD-NC, in 50% (81 patients) of PD-MCI, and in 42% (22 patients) of PDD. Results are reported in details in Table 2 and Figure 2.

**Table 2:** Severity and frequencies of ICDs and ICBs across cognitive states.

	**PD-NC (*****n*** **=** **110)**			**PD-MCI (*****n*** **=** **163)**			**PDD (*****n*** **=** **53)**			**ANCOVA**
	**QUIP-RS score** ** Mean (SD)^a^**	**% QUIP-RS>0^b^**	**% QUIP-RS above cutoff^b^**	**QUIP-RS score** ** Mean (SD)^a^**	**% QUIP-RS>0^b^**	**% QUIP-RS above cutoff^b^**	**QUIP-RS score** ** Mean (SD)^a^**	**%QUIP-RS>0^b^**	**% QUIP-RS above cutoff^b^**	***P-*** **value**
**ICD+** **(MIDI)**	10.56 (8.90)	55		10.19 (7.74)	50		10.41 (8.48)	42		0.877
**ICD**^c^	4.00 (1.65)	22	0	4.85 (3.02)	20	2	3.66 (2.25)	21	2	0.769
PG only		1	0		2	0		0	0	
HS only		5	0		4	0		8	0	
CS only		8	0		3	0		2	0	
BE only		5	0		4	0		6	0	
Multiple ICD		3	0		7	2		5	2	
**ICB**^c^	4.73 (3.17)	10	4	5.64 (4.14)	9	2	/	0	0	0.329
Hobbyism only		7	3		4	1		0	0	
Punding only		3	1		0	0		0	0	
DDS only		0	0		3	1		0	0	
Multiple ICB		0	0		1	0		0	0	
**ICD & ICB**^c^	17.33 (8.40)	22	20	16.49 (6.96)	20	20	14.09 (8.38)	21	21	0.976

ICD+, impulse control disorders and related behaviors according to MIDI; ICD, patients with ICD but not ICB; ICB, patients with ICB but not ICD; Multiple ICD, patients with multiple ICDs but no ICB; Multiple ICB, patients with multiple ICBs but no ICD; ICD&ICB, patients with at least an ICD and an ICB; PG, pathological gambling; HS, hypersexuality; CS, compulsive shopping; BE, binge-eating; DDS, dopamine dysregulation syndrome; PD, Parkinson's disease; PD-NC, PD with normal cognition; PD-MCI, PD with mild cognitive impairment; PDD, PD with dementia; QUIP-RS, Questionnaire for Impulsive-Compulsive Disorders in Parkinson's Disease-Rating Scale; % QUIP-RS>0, percentage of patients with QUIP-RS score above 0; % QUIP-RS above cutoff, percentage of patients with QUIP-RS score above the cutoffs.

a No significant differences were found on severity (QUIP-RS mean scores) between PD subgroups after ANCOVA including disease duration, DAED, UPDRS-I, ADL, PDQ-8, BDI-II and MoCA scores as covariates.

b No significant differences or linear trends were found within and between PD subgroups.

c *Percentages of patients with single ICDs, single ICBs, multiple ICDs, multiple ICBs, or at least an ICD and an ICB (ICD&ICB), according to previously published cutoff values (PG: QUIP-RS ≥ 6; HS: QUIP-RS ≥ 8; CS: QUIP-RS ≥ 8; BE: QUIP-RS ≥ 7; hobbyism: QUIP-RS ≥ 7; punding: QUIP-RS ≥ 7; DDS: QUIP-RS ≥ 7; multiple ICDs: QUIP-RS combined ICDs score ≥ 10; multiple ICBs: QUIP-RS combined ICBs score ≥ 7) (30)*.

Frequencies decrease across cognitive states, but trend toward a decrease of frequencies with cognitive decline do not reach statistical significance (*p* = 0.34).

According to the QUIP-RS, either ICDs or ICBs above the cutoff were present in the 24% of PD-NC, in the 24% of PD-MCI, and in the 23% of PDD. The 20% of PD-NC, the 20% of PD-MCI, and the 21% of PDD, presented both ICDs and ICBs.

Considering QUIP-RS scores above 0, either ICDs or ICBs were present in the 54% of PD-NC, in the 49% of PD-MCI, and in 42% of PDD.

Severity of ICD+ did not differ across cognitive states (*p* = 0.877). No differences were also observed considering ICDs and ICBs separately (*p* = 0.769 and *p* = 0.329, respectively) (see Table 2).

### ICDs and Cognitive Phenotypes

In the PD-NC group, there were no differences between the percentages of ICD+ and ICD– failing two tests of the same cognitive domain.

In PD-MCI, there was higher number of ICD+ patients failing two tests of attention (ICD+: 20% vs. ICD–: 4%; *p* = 0.031) (see Table 3). Percentage of patients with TMT B-A z-scores below 1.5 SD was significantly higher in the ICD+ than in the ICD– subgroup (ICD+: 41%; ICD–: 24%; *p* = 0.035), with no differences in the DSS performances (see Table 4).

**Table 3:** Frequencies of patients with a failure in at least two tests within a cognitive domain across cognitive states and ICD-subgroups.

**Cognitive domains**	**PD-MCI** ** (*****n*** **=** **147)**		**PDD** ** (*****n*** **=** **49)**		**PD-MCI**	**PDD**
	**ICD–** ** (*n* = 75) (%)**	**ICD+** ** (*n* = 72) (%)**	**ICD–** ** (*n* = 29) (%)**	**ICD+** ** (*n* = 20) (%)**	***P*****-value** ** ICD+** **vs. ICD-**	
Attention/working memory	4	20	76	73	**0.0315**	0.8453
Executive	5	4	30	44	0.8550	**0.0279**
Language	3	0	26	36	0.4970	0.8687
Memory	2	9	48	47	0.2246	0.7930
Visuospatial	22	27	80	81	0.7241	0.7138

*Significant differences (p < 0.05) are reported in bold type. PD, Parkinson's disease; PD-NC, PD with normal cognition; PD-MCI, PD with mild cognitive impairment; PDD, PD with dementia; ICD+, patients with impulse control disorders and related behaviors according to MIDI; ICD-, patients without impulse control disorders and related behaviors according to MIDI*.

**Table 4:** Percentage of ICD+ and ICD– across cognitive states with a cognitive performance below 1.5 SD, in the attentive and executive domains.

	**PD-MCI**		**PDD**		**PD-MCI**	**PDD**
	**ICD– (%)**	**ICD+ (%)**	**ICD– (%)**	**ICD+ (%)**	***P-*****value**	
**ATTENTION AND WORKING MEMORY DOMAIN**						
TMT B-A	24	41	79	60	**0.0350**	0.2500
DSS (WAIS-IV)	13	15	58	75	0.9190	0.3790
**EXECUTIVE DOMAIN**						
Stroop test	46	49	75	89	0.9141	0.4609
Similarities	15	18	29	67	0.8948	**0.0355**

*Significant differences (p < 0.05) are reported in bold type. PD, Parkinson's disease; PD-NC, PD with normal cognition; PD-MCI, PD with mild cognitive impairment; PDD, PD with dementia; ICD+, patients with impulse control disorders and related behaviors according to MIDI; ICD–, patients without impulse control disorders and related behaviors according to MIDI; TMT B-A, Trail Making Test part B-A; DSS, Digit Span Sequencing subtest of the Wechsler Adult Intelligence Scale–Fourth Edition; SD, standard deviation*.

In PDD, there were higher rates of ICD+ patients failing two tests of executive function (ICD+: 44% vs. ICD–: 30%; *p* = 0.027), with no differences in the other domains (see Table 3). Data seems to be driven by the Similarities task as the percentage of patients with z-scores below 1.5 SD was significantly higher in the ICD+ than in the ICD– subgroup (ICD+: 67%; ICD–: 29%; *p* = 0.035), with no differences in the Stroop task (see Table 4).

Detailed demographic characteristics of PD-MCI group based on performances at TMT B-A test and PDD based on performance at Similarities task are provided in the Supplementary Tables 2, 3.

## Discussion

This is the first study describing prevalence and characteristics of ICDs and related behaviors in PD cognitive states including both PD with dementia and PD-MCI. We found that their prevalence tends to decrease from PD-NC to PDD, although differences in rates were not significant while severity was similar across cognitive states.

These findings are different from other studies reporting an association with cognitive performance (14, 15) and particularly with one prevalence study in which ICDs were less frequent in PDD compared to PD-NC (26). Discrepancies with the latter study, may reflect differences in PDD diagnostic procedures (46). In our cohort all patients underwent level II cognitive, daily functioning and behavioral assessments, and cognitive states diagnosis included PD-MCI as well as PD-NC and PDD, following proposed criteria for PD (28, 29).

Indeed clinical and demographic characteristics in our cohort of PD-NC, PD-MCI, and PDD are in line with literature, and this was indirectly confirmed by the observation of older age, longer disease duration, worse motor symptomatology, cognitive decline, and depression levels in our PDD (16, 47, 48).

In our study, diagnosis of ICDs or ICBs was based on the MIDI and behaviors that were not included in the MIDI but commonly occur in PD were also investigated. The QUIP-RS, since it has not been validated in the Italian population, was used only for assessing severity. In order to characterize the type of ICDs and ICBs of our sample, data were also presented according to published US sample cutoff score (30) further validated in the German population (49). According to published cutoff scores (30), pure single ICDs were not present in any patient in our cohort. This may imply either that QUIP-RS cutoff scores are too conservative for Italian population, or that ICDs infrequently occur as single entity. In any case, future studies are needed to further explore this point.

Exploring ICDs frequency based on scores of QUIP-RS>0, we found similar results. Of note, frequencies of HS and BE were similar in PDD and in PD-NC regardless of lower DAED levels and lower number of patients on dopamine agonists (8, 23, 24, 50, 51). We speculate that similar rates might be either due to (i) shared underlying mechanisms (i.e., dementia-like neurodegenerations vs. ICDs-related) or (ii) the characteristics of QUIP-RS, which may capture features of disinhibitions related to impulsivity without ruling out dementia-like behavioral disinhibition (50, 52).

Our study confirms, in PD-NC and PD-MCI, previously reported risk factors for ICD. In the PD-NC group, ICD was associated with higher disease duration and LEDD. In the PD-MCI, ICD was associated with lower age and age at symptoms onset, and higher disease duration, LEDD, DAED, and percentage of patients under DA. Conversely, ICD+ and ICD– PDD patients did not differ in any demographic and clinical variable investigated. For a clinical point of view, these finding suggest that i) ICD are equally common in PDD as PD-NC and PD-MCI, and that ii) the recognized risk factors for ICD in PD may not apply to PDD, further encouraging physician awareness.

Furthermore, quality of life, as assessed by PDQ-8, differs between ICD+ and ICD- in PD-NC and PD-MCI as previously reported (13). Interestingly, we do not find any difference in PDQ-8 score of PDD patients with and without ICDs maybe because other motor and/or non-motor symptoms are likely to impact more than ICDs on QoL.

Despite frequencies and severities of ICDs were similar across PD-NC, PD-MCI and PDD, patterns of cognitive alterations (i.e., failure in two tests of the same domain), associated with presence/absence of ICDs, differed within each cognitive state. Presence of ICDs in PD-MCI is associated with attention impairments, whilst in PDD with ICDs cognitive decline involved the executive domain. In PD-NC, there were no patterns of cognitive alterations and this reflects the MDS guidelines, with failure in two tests of the same cognitive domain indicative of PD-MCI (28). Taken together these findings support frontal-striatal (i.e., executive and attentive) instead of posterior impairments (i.e., language and visuospatial abilities) in ICD+ (53–55) and the involvement of altered mesocorticolimbic activity (56–58). Moreover, this study further extends previous results showing that the patterns of frontal dysfunctions of ICD+ differ within each cognitive state. Clinically, these results have important implications as attentive impairments in PD-MCI and executive dysfunctions in PDD measured by level II neuropsychological assessment may suggest co-presence of ICDs and related behaviors.

When performances were analyzed considering the single neuropsychological test, the TMT B-A but not the DSS was associated with higher rates of ICD+ in PD-MCI. Worse TMT-B-A performances have been reported in non-PD pathological gamblers (59) and in PD patient with ICDs (53, 60, 61), although no specifically investigated within cognitive states. The TMT B-A and the DSS, albeit being categorized within the attentive domain are tasks investigating set-shifting and working memory abilities, respectively. TMT B-A requires cognitive flexibility in order to switch from numerical to alphabetical sequences, which is an important ability for maintaining goal-oriented behaviors when facing environmental changes or task demands in daily life (62). In lesion mapping studies, TMT B-A performances are associated with rostral anterior cingulate cortex (63), which is part of the mesocorticolimbic pathway mediating the control of reward-related behaviors that may be overstimulated by dopaminergic medication. In the early stages of the PD, dopaminergic depletion is relatively circumscribed to the dorsal striatum, whilst the limbic (nucleus accumbens) and cortical (prefrontal cortex) structures are relatively spared and only degenerate in the later stages (64). Medication levels necessary to restore dopaminergic depletion in the dorsal striatum may abnormally stimulate mesocorticolimbic structures. Interestingly, deficits in the TMT B-A task are more common in PD-MCI patients with lower age and lower age at symptoms onset, longer disease duration, higher DEAD and LEDD levels, and higher percentage of dopamine agonists use (see Supplementary Table 2) who may be more vulnerable to the overdosing effect of medication. The TMT B-A, albeit being a sensitive test of ICD+ in PD-MCI, may not be indicated for assessing PDD patients. In our sample, high number of PDD patients was not able to perform either the TMT B-A or the DDS.

In PDD patients, performance in the Similarities but not the Stroop tasks was associated with ICD+. Lack of differences between ICD+ and ICD– patients in the Stroop task (2, 53, 54, 65, 66) as well as in the Similarities task (53, 65) have been reported, although in these studies dementia was an exclusion criteria. This may explain why we found that ICD+ was associated with impairments in the Similarities task contrarily the previous results (53, 65). Compared to the Stroop task that evaluates verbal inhibition, the similarities task assesses abstract thinking, concept formation, and verbal reasoning as participants are instructed to describe how two things are similar. Abstract thinking is associated with anterior prefrontal, fronto-parietal cortices, and insula functioning (63). Therefore, we might speculate that, as PD cognitive severity increases, presence of ICDs is associated with wider cortical and subcortical dysfunctions which target limbic and frontal and parietal areas. PDD patients who fail the Similarities task present worse general cognitive performance and higher levels of trait anxiety (see Supplementary Table 3).

Although the study was conducted in a large cohort of PD patients following proposed guidelines for PD-MCI and PDD diagnosis, there are some limitations that should be acknowledged.

First, participants were recruited during clinics and this limits the generalizability of the results to the whole PD population. Second, the QUIP-RS has not been validated in the Italian population therefore prevalence rates of ICDs according to QUIP-RS cutoff scores should be considered cautiously as may not apply for our sample. However, patients were categorized as ICD+ by an experiencing neuropsychologist who also administered the MIDI and clinical diagnosis was done according to established diagnostic criteria. Third, in PDD, lack of differences between ICD+ and ICD- in the attentive domain might be biased by the floor effect of the TMT B-A and DSS, with high number of PDD patients not able to perform the tasks. Fourth, the TMT B-A and Similarities tasks are not purely attentive and executive, but they also investigate executive functions and language, respectively (67). However, we might exclude a language involvement in PDD with ICD as performances in semantic fluencies and naming did not differ between ICD+ and ICD–. Further studies should use experimental tasks investigating specific cognitive processes to assess neurological underpinnings of ICDs and medication effects across cognitive states and domains.

In conclusion, our findings provide evidence that cognitive states *per sè* are not associated with (i) the presence and the (ii) severity of ICDs and related behaviors. Conversely, (iii) impairments in ICD+ are circumscribed to attentive and executive domains in PD-MCI and PDD patients, respectively. Finally, (iv) the TMT B-A task for the attention and working memory domain in PD-MCI, and the Similarities task for the executive function domain in the PDD were the tasks more sensitive of ICD and related behavior presence. Taken together these findings may suggest different ICDs entities according to disease cognitive progression. Namely, a relative early phase dopamine agonist dependent ICDs characterized by mainly attentive problems and a late phase medication independent ICDs characterized by wider cortical and dysexecutive dysfunctions. Future studies should help addressing this hypothesis.

PD patients should be carefully interviewed for the presence of ICDs and related behaviors at any stage of the disease, as being diagnosed either with PD-MCI, PDD, or being PD-NC is not indicative *per sè* of a higher or lower risk of ICD.

## Data Availability

The datasets for this study will not be made publicly available because the authors don't have the permission to share the dataset.

## Author Contributions

The study has been designed by RB, LW, and AA. Data have been collected by RS, VC, and EF and analyzed by LW. The manuscript has been drafted by AM, EF, and RB. AM, LW, EF, RS, VC, AA, and RB revised the manuscript.

### Conflict of Interest Statement

The authors declare that the research was conducted in the absence of any commercial or financial relationships that could be construed as a potential conflict of interest.
