# Supplementary material for: Impact of Cognitive Profile on Impulse Control Disorders Presence and Severity in Parkinson's Disease
Source: Front Neurol. 2019 Mar 22;10:266. doi: 10.3389/fneur.2019.00266 (PMC6439312; doi:10.3389/fneur.2019.00266)
Supplement: Supplementary file 1 [file Table_1.DOCX]

**Supplementary Table 1** Demographical and clinical characteristics across PD cognitive states

|  | **PD-NC**  **(n = 110)** |  | **PD-MCI**  **(n = 163)** |  | **PDD**  **(n = 53)** |  | **Kruskal–Wallis H test** | **Post-hoc** | | |  |
| --- | --- | --- | --- | --- | --- | --- | --- | --- | --- | --- | --- |
|  | Mean (SD) | | | | |  | *P value* | PD-NC vs.  PD-MCI | PD-NC vs. PDD | PD-MCI vs.  PDD | |
|  |  |  |  |  |  |  |  |  |  |  |  |
| **Age** (yr) | 60.68 (9.66) |  | 68.26 (9.23) |  | 72.87 (8.28) |  | **<0.0001** | **x** | **x** | **x** |  |
| **Sex** (%, male) | 54% |  | 62% |  | 69% |  | 0.1250 |  |  |  |  |
| **Education** (yr) | 12.81 (3.93) |  | 9.64 (4.41) |  | 9.26 (4.71) |  | **<0.0001** | **x** | **x** |  |  |
| **Age of onset symptoms** (yr) | 52.23 (10.35) |  | 58.33 (11.86) |  | 61.47 (9.92) |  | **<0.0001** | **x** | **x** |  |  |
| **Disease duration** (yr) | 8.23 (5.23) |  | 9.25 (6.34) |  | 11.2 (5.01) |  | **0.0066** |  | **x** | **x** |  |
| **LEDD** | 847.21 (526.08) |  | 875.98 (517.29) |  | 722.12 (393.53) |  | 0.1840 |  |  |  |  |
| **LEDD/kg** | 12.28 (8.26) |  | 12.36 (7.64) |  | 9.68 (5.18) |  | 0.1170 |  |  |  |  |
| **DA** (%) | 80% |  | 80% |  | 54% |  | **0.0005** |  | **x** | **x** |  |
| **DAED** | 156.45 (117.28) |  | 129.99 (104.41) |  | 76.83 (89.27) |  | **0.0002** |  | **x** | **x** |  |
| **DAED/kg** | 2.26 (1.79) |  | 1.85 (1.61) |  | 1.02 (1.17) |  | **0.0001** |  | **x** | **x** |  |
| **MDS-UPDRS-I** | 10.49 (5.26) |  | 10.36 (4.64) |  | 16.44 (7.58) |  | **0.0002** |  | **x** | **x** |  |
| **MDS-UPDRS-II** | 11.08 (6.23) |  | 12.75 (6.84) |  | 19.68 (7.26) |  | **<0.0001** |  | **x** | **x** |  |
| **MDS-UPDRS-III** | 19.68 (12.32) |  | 25.92 (12.41) |  | 35.17 (12.11) |  | **<0.0001** | **x** | **x** | **x** |  |
| **ADL** | 5.75 (0.68) |  | 5.42 (0.96) |  | 3.89 (1.74) |  | **<0.0001** |  | **x** | **x** |  |
| **IADL** | 5.99 (1.52) |  | 5.50 (1.64) |  | 3.06 (1.7) |  | **<0.0002** |  | **x** | **x** |  |
| **PD-CFRS** | 1.97 (2.25) |  | 4.11 (3.8) |  | 12.66 (6.35) |  | **<0.0001** | **x** | **x** | **x** |  |
| **PDQ-8** | 8.07 (5.2) |  | 9.56 (5.5) |  | 13.38 (5.81) |  | **<0.0001** | **x** | **x** | **x** |  |
| **STAI-Y1** | 37.72 (10.4) |  | 38.77 (10.22) |  | 43.24 (9.6) |  | **0.0076** |  | **x** | **x** |  |
| **STAI-Y2** | 41.53 (10.45) |  | 41.19 (10.34) |  | 45.08 (10.67) |  | 0.1660 |  |  |  |  |
| **BDI-*II*** | 9.16 (8.01) |  | 10.56 (7.58) |  | 14.13 (7.18) |  | **<0.0001** | **x** | **x** | **x** |  |
| **BDI-*II*** (%, cutoff > 14) | 19% |  | 27% |  | 46% |  | **0.0027** |  | **x** | **x** |  |
| **MoCA** | 25.54 (2.53) |  | 22.2 (3.31) |  | 16.63 (4.13) |  | **<0.0001** | **x** | **x** | **x** |  |
| **MMSE** | 27.63 (2.53) |  | 25.75 (2.5) |  | 21.24 (4.22) |  | **<0.0001** | **x** | **x** | **x** |  |

*Note.* Significant differences (p < 0.05) are reported in bold type. SD, standard deviation; PD, Parkinson’s disease; PD-NC, PD with normal cognition; PD-MCI, PD with mild cognitive impairment; PDD, PD with dementia; MDS-UPDRS, Movement Disorder Society Unified Parkinson’s Disease Rating Scale; LEDD, levodopa equivalent daily dose; DAED, dopamine agonist equivalent dose; LEDD/kg, LEDD adjusted by body weight; DAED/kg, DAED adjusted by body weight; ADL, Activity of daily living; IADL, Instrumental activities of daily living; PD-CFRS, Parkinson's Disease - Cognitive Functional Rating Scale; PDQ-8, Parkinson's Disease Questionnaire; STAI (Y-1, Y-2), State-Trait Anxiety Inventory; BDI-*II*, Beck Depression Inventory-*II*; MoCA, Montreal Cognitive Assessment; MMSE, Mini Mental State Examination.
