# Supplementary material for: Impact of Cognitive Profile on Impulse Control Disorders Presence and Severity in Parkinson's Disease
Source: Front Neurol. 2019 Mar 22;10:266. doi: 10.3389/fneur.2019.00266 (PMC6439312; doi:10.3389/fneur.2019.00266)
Supplement: Supplementary file 2 [file Table_2.docx]

**Supplementary Table 2** Demographical and clinical characteristics across PD-MCI based on performances in TMT B-A

|  | **PD MCI** | | | | Mann Whitney U test |
| --- | --- | --- | --- | --- | --- |
|  | **TMT B-A**  ***z*-score > 1.5 SD** | | **TMT B-A**  ***z*-score < 1.5 SD** | |  |
|  | Mean | SD | Mean | SD |  |
| **Age** (yr) | 69.65 | 8.69 | 65.76 | 9.19 | **0.0027** |
| **Sex** (%, male) | 63% |  | 60% |  | 0.8467 |
| **Education** (yr) | 10.13 | 4.68 | 8.73 | 3.67 | 0.0890 |
| **Age of onset symptoms** (yr) | 60.78 | 11.19 | 53.45 | 11.58 | **0.0001** |
| **Disease duration** (yr) | 8.41 | 5.77 | 11.26 | 7.13 | **0.0140** |
| **LEDD** | 778.48 | 486.77 | 1098.53 | 521.44 | **0.0004** |
| **LEDD/kg** | 11.14 | 7.31 | 15.12 | 7.76 | **0.0026** |
| **DA** (%) | 74% |  | 92% |  | **0.0130** |
| **DAED** | 115.14 | 105.46 | 162.6 | 95.92 | **0.0029** |
| **DAED/kg** | 1.67 | 1.69 | 2.23 | 1.37 | **0.0025** |
| **MDS-UPDRS-I** | 10.59 | 4.85 | 9.97 | 4.31 | 0.7550 |
| **MDS-UPDRS-II** | 12.63 | 6.56 | 12.97 | 7.51 | 0.9540 |
| **MDS-UPDRS -III** | 25.08 | 10.62 | 27.67 | 15.37 | 0.6590 |
| **STAI-Y1** | 38.68 | 10.11 | 39.10 | 10.68 | 0.8160 |
| **STAI-Y2** | 40.95 | 10.27 | 41.71 | 10.35 | 0.6560 |
| **ADL** | 5.44 | 0.90 | 5.37 | 1.10 | 0.9520 |
| **IADL** | 5.57 | 1.64 | 5.37 | 1.65 | 0.3800 |
| **BDI-*II*** | 10.20 | 7.44 | 11.42 | 7.84 | 0.2580 |
| **PDQ-8** | 9.10 | 5.47 | 10.60 | 5.42 | 0.5190 |
| **MoCA** | 26.17 | 2.11 | 24.95 | 3.02 | 0.0630 |
| **MMSE** | 4.03 | 3.95 | 4.29 | 3.58 | **0.0073** |
| **ICD (% above cutoff)** |  |  |  |  |  |
| **Gambling** | 2.60% |  | 7.40% |  | 0.3074 |
| **Hypersexuality** | 6.25% |  | 3.70% |  | 0.7540 |
| **Shopping** | 0.80% |  | 3.70% |  | 0.5140 |
| **Binge-eating** | 8.50% |  | 12.00% |  | 0.7000 |
| **Hobbyism** | 4.80% |  | 2.00% |  | 0.6900 |
| **Punding** | 0.94% |  | 4.00% |  | 0.5000 |
| **DDS** | 0.96% |  | 2.00% |  | 0.8200 |

*Note.* Significant differences (p < 0.05) are reported in bold type. TMT B-A, Trail Making Test part B-A; SD, standard deviation; PD, Parkinson’s disease; PD-MCI, PD with mild cognitive impairment MDS-UPDRS, Movement Disorder Society Unified Parkinson’s Disease Rating Scale; LEDD, levodopa equivalent daily dose; DAED, dopamine agonist equivalent dose; LEDD/kg, LEDD adjusted by body weight; DAED/kg, DAED adjusted by body weight; ADL, Activity of daily living; IADL, Instrumental activities of daily living; PDQ-8, Parkinson's Disease Questionnaire; STAI (Y1, Y2), State-Trait Anxiety Inventory; BDI-*II*, Beck Depression Inventory-*II*; MoCA, Montreal Cognitive Assessment; MMSE, Mini Mental State Examination; ICD, impulsive compulsive disorder; DSS, dopamine dysregulation syndrome.
