# Supplementary material for: Impact of Cognitive Profile on Impulse Control Disorders Presence and Severity in Parkinson's Disease
Source: Front Neurol. 2019 Mar 22;10:266. doi: 10.3389/fneur.2019.00266 (PMC6439312; doi:10.3389/fneur.2019.00266)
Supplement: Supplementary file 3 [file Table_3.docx]

**Supplementary Table 3** Demographical and clinical characteristics across PDD based on performances in Similarities (WAIS-IV)

|  | **PDD** | | | | Mann Whitney U test |
| --- | --- | --- | --- | --- | --- |
|  | **Similarities**  ***z-*score > 1.5 SD** | | **Similarities**  ***z-*score < 1.5 SD** | |  |
|  | Mean | SD | Mean | SD |  |
| **Age** (yr) | 73.68 | 8.55 | 71.68 | 9.46 | 0.4980 |
| **Sex** (%, male) | 72% |  | 61% |  | 0.6730 |
| **Education** (yr) | 10.12 | 4.85 | 8.32 | 4.31 | 0.2010 |
| **Age of onset symptoms** (yr) | 63.32 | 10.96 | 60.17 | 9.18 | 0.3900 |
| **Disease duration** (yr) | 10.36 | 5.1 | 11.5 | 4.87 | 0.4690 |
| **LEDD** | 619.42 | 327.46 | 819.85 | 415.76 | 0.1440 |
| **LEDD/kg** | 8.35 | 4.16 | 11.01 | 5.51 | 0.1904 |
| **DA** (%) | 48% |  | 61% |  | 0.6030 |
| **DAED** | 57.6 | 79.21 | 97.61 | 97.46 | 0.2020 |
| **DAED/kg** | 0.76 | 1.04 | 1.29 | 1.24 | 0.1820 |
| **MDS-UPDRS-I** | 17.92 | 8.36 | 15.58 | 6.87 | 0.7490 |
| **MDS-UPDRS-II** | 19.75 | 8.88 | 20.33 | 5.37 | 0.7730 |
| **MDS-UPDRS -III** | 33.15 | 12.83 | 36.93 | 12.34 | 0.5270 |
| **STAI-Y1** | 42.59 | 9.51 | 46 | 10.72 | 0.4270 |
| **STAI-Y2** | 41.82 | 8.15 | 50.75 | 13.12 | **0.0250** |
| **ADL** | 4 | 1.93 | 3.94 | 1.69 | 0.7490 |
| **IADL** | 3.39 | 1.37 | 2.88 | 1.96 | 0.4090 |
| **BDI-*II*** | 13.39 | 6.58 | 14.59 | 6.96 | 0.4590 |
| **PDQ-8** | 13.35 | 5.7 | 13.13 | 6.03 | 0.9730 |
| **MoCA** | 16.99 | 4.04 | 15.66 | 3.66 | 0.2500 |
| **MMSE** | 22.58 | 3.63 | 19.85 | 4.7 | **0.0500** |
| **ICD (% above cutoff)** |  |  |  |  |  |
| **Gambling** | 0% |  | 11% |  | 0.3520 |
| **Hypersexuality** | 8% |  | 16% |  | 0.7440 |
| **Shopping** | 0% |  | 5% |  | 0.8890 |
| **Binge-eating** | 0% |  | 13% |  | 0.3160 |
| **Hobbyism** | 0% |  | 6% |  | 0.8530 |
| **Punding** | 0% |  | 0% |  | 1.0000 |
| **DDS** | 0% |  | 0% |  | 1.0000 |

*Note.* Significant differences (p < 0.05) are reported in bold type. WAIS–IV Wechsler Adult Intelligence Scale–Fourth Edition; SD, standard deviation; PD, Parkinson’s disease; PDD, PD with dementia; MDS-UPDRS, Movement Disorder Society Unified Parkinson’s Disease Rating Scale; LEDD, levodopa equivalent daily dose; DAED, dopamine agonist equivalent dose; LEDD/kg, LEDD adjusted by body weight; DAED/kg, DAED adjusted by body weight; ADL, Activity of daily living; IADL, Instrumental activities of daily living; PDQ-8, Parkinson's Disease Questionnaire; STAI (Y1, Y2), State-Trait Anxiety Inventory; BDI-*II*, Beck Depression Inventory-*II*; MoCA, Montreal Cognitive Assessment; MMSE, Mini Mental State Examination; ICD, impulsive compulsive disorder; DSS, dopamine dysregulation syndrome.
